# Population Differentiation of Southern Indian Male Lineages Correlates with Agricultural Expansions Predating the Caste System

**DOI:** 10.1371/journal.pone.0050269

**Published:** 2012-11-28

**Authors:** GaneshPrasad ArunKumar, David F. Soria-Hernanz, Valampuri John Kavitha, Varatharajan Santhakumari Arun, Adhikarla Syama, Kumaran Samy Ashokan, Kavandanpatti Thangaraj Gandhirajan, Koothapuli Vijayakumar, Muthuswamy Narayanan, Mariakuttikan Jayalakshmi, Janet S. Ziegle, Ajay K. Royyuru, Laxmi Parida, R. Spencer Wells, Colin Renfrew, Theodore G. Schurr, Chris Tyler Smith, Daniel E. Platt, Ramasamy Pitchappan, The Genographic Consortium

**Affiliations:** 1 The Genographic Laboratory, School of Biological Sciences, Madurai Kamaraj University, Madurai, Tamil Nadu, India; 2 National Geographic Society, Washington, District of Columbia, United States of America; 3 Institut de Biologia Evolutiva (CSIC-UPF), Departament de Ciències Experimentals i de la Salut, Universitat Pompeu Fabra, Barcelona, Spain; 4 Department of Biotechnology, Mother Teresa Women's University, Kodaikanal, Tamil Nadu, India; 5 Nilgiri Adivasi Welfare Association, Kota Hall Road, Kothagiri, Tamil Nadu, India; 6 Government College of Fine Arts, Chennai, Tamil Nadu, India; 7 Department of Zoology, St. Xaviers College, Palayamkottai, Tamil Nadu, India; 8 Applied Biosystems, Foster City, California, United States of America; 9 Computational Biology Group, IBM - Thomas J. Watson Research Center, New York, New York, United States of America; 10 McDonald Institute for Archaeological Research, University of Cambridge, Cambridge, United Kingdom; 11 Department of Anthropology, University of Pennsylvania, Philadelphia, Pennsylvania, United States of America; 12 The Wellcome Trust Sanger Institute, Wellcome Trust Genome Campus, Hinxton, United Kingdom; 13 Chettinad Academy of Research and Education, Kelampakkam, Chennai, Tamil Nadu, India; Erasmus University Medical Center, The Netherlands

## Abstract

Previous studies that pooled Indian populations from a wide variety of geographical locations, have obtained contradictory conclusions about the processes of the establishment of the Varna caste system and its genetic impact on the origins and demographic histories of Indian populations. To further investigate these questions we took advantage that both Y chromosome and caste designation are paternally inherited, and genotyped 1,680 Y chromosomes representing 12 tribal and 19 non-tribal (caste) endogamous populations from the predominantly Dravidian-speaking Tamil Nadu state in the southernmost part of India. Tribes and castes were both characterized by an overwhelming proportion of putatively Indian autochthonous Y-chromosomal haplogroups (H-M69, F-M89, R1a1-M17, L1-M27, R2-M124, and C5-M356; 81% combined) with a shared genetic heritage dating back to the late Pleistocene (10–30 Kya), suggesting that more recent Holocene migrations from western Eurasia contributed <20% of the male lineages. We found strong evidence for genetic structure, associated primarily with the current mode of subsistence. Coalescence analysis suggested that the social stratification was established 4–6 Kya and there was little admixture during the last 3 Kya, implying a minimal genetic impact of the Varna (caste) system from the historically-documented Brahmin migrations into the area. In contrast, the overall Y-chromosomal patterns, the time depth of population diversifications and the period of differentiation were best explained by the emergence of agricultural technology in South Asia. These results highlight the utility of detailed local genetic studies within India, without prior assumptions about the importance of Varna rank status for population grouping, to obtain new insights into the relative influences of past demographic events for the population structure of the whole of modern India.

## Introduction

Contemporary Indian populations exhibit a high cultural, morphological, and linguistic diversity, as well as some of the highest genetic diversities among continental populations after Africa [1], [2]. Indian populations are broadly classified into two categories: ‘tribal’ and ‘non-tribal’ groups [3]. Tribal groups, constituting 8% of the Indian population, are characterized by traditional modes of subsistence such as hunting and gathering, foraging and seasonal agriculture of various kinds [2], [3]. In contrast, most other Indians fall into non-tribal categories, many of them classified as castes under the Hindu Varna (Color caste) system which groups caste populations, primarily on occupation, into Brahmin (priestly class), Kshatriya (warrior and artisan), Vyasa (merchant), Shudra (unskilled labor) and the most recently added fifth class, Panchama, the scheduled castes of India [2], [3]. Generally, both non-tribal and tribal populations employ a patrilineal caste endogamy. This practice, together with the male-specific genetic transmission of the non-recombining portion of the Y-chromosome (NRY), provides a unique opportunity to study the impact of historical demographic processes and the social structure on the gene pool of India.

The distribution of deep-rooted Indian-specific Y-chromosomal and mitochondrial lineages suggests an initial settlement of modern humans in the subcontinent from the early out-of-Africa migration [4], [5], [6], [7], [8], [9]. The greater genetic isolation of many tribal groups and their differences in Y-chromosomal haplogroup (HG) lineages compared to non-tribal groups, have generally been interpreted as evidence of tribes being direct descendants of the earliest Indian settlers [2], [10], [11], [12], [13]. Moreover, these tribe-caste genetic differences have been attributed to the establishment of the Hindu Varna system that has been maintained for millennia since both Y chromosome and caste designation are paternally inherited. However, the origin of caste system in India is still a controversial subject [8], [14], [15], [16], and there are two main schools of thought about it. First, demic diffusion models propose an expansion of Indo-European (IE) speakers 3 Kya (thousand years ago) from Central Asia [10], [17], [18], [19], [20], [21], [22]. Alternatively, other models propose the origin of caste as the result of cultural diffusion and/or autochthonous demographic processes without any major genetic influx from outside India [6], [7], [16], [23]. Overall, the genetic impact and mode of establishment of the caste system, the extent of a common indigenous Pleistocene (10 Kya to 30 Kya) genetic heritage and the degree of admixture from West Eurasian Holocene (10 Kya) migrations and their level of impact on the tribal and non-tribal groups from India, remain unresolved [5], [6], [7], [10], [16].

The lack of consensus among previous studies may reflect difficulties associated with the conflicting relationships between genetics and the socio-cultural factors used to pool truly endogamous groups into broader categories, sometimes grouping Indian populations sampled from a wide variety of geographical locations together, such as a tribe-caste dichotomy or caste-rank hierarchy [2], [5], [7]. One goal of pooling data from multiple populations has been to smooth individual drift effects in an effort to reconstruct putative ancestry [10] and thereby potentially infer the past demographic processes shaping genetic diversity. However, the success of this approach relies on whether the classification employed indeed reflects the true historical relationships among these endogamous groups. Methods seeking to identify the best grouping from an exploration of alternative possible classifications, based on seeking maximal between-population differences and minimal within-population variation [24], would be of special relevance for studies on Indian populations classified based on Varna status. This is the case because several castes have suffered from historically fluid definitions of their rank status, and both the origins and the scope of the genetic impact of the Varna system on these populations are still unclear [8], [20], [25], [26], [27], [28]. Further, since the implementation of the Varna system throughout India was not a uniform process [17], broad classifications of multiple Indian samples from all over the subcontinent based on Varna status, or tribe-caste dichotomy, may not reflect true endogamous populations and could also obscure genetic signals and the finer details of Indian demographic histories. For this reason, a genetic study using a careful and extensive sampling of well-defined non-tribal and tribal endogamous populations from a restricted area designed to reduce the confounding relationships among socio-cultural factors, without presuming Varna rank status, to find empirically the best approach of population grouping, could be a successful model to obtain new insights of past Indian demographic processes.

Here, we attempted to apply this strategy to unravel the population structure and genetic history of the southernmost state of India, Tamil Nadu (TN), which is well known for its rigid caste system [15], and to relate the resulting genetic data to the paleoclimatic, archaeological, and historical evidence from this region. The paleoclimatic and archaeological records show post-LGM (Last Glacial Maximum) wet period expansions of foragers into the region, whose interactions with later aridification-driven migrations of agriculturists have been traced [29], [30], [31], [32], [33], [34], [35]. Archaeology also reveals the establishment of metallurgy [36] and river settlements [17], just several centuries prior to the creation of the earliest written records of the Sangam literature (300 BCE to 300 CE). These historical records named several populations including some in the present study (e.g., Paliyan, Pulayar, Valayar) reflecting the existence of these now endogamous groups at that time [37], [38]. More recent reports dated to the 6^th^ century CE, under the reign of the Sarabhapuriyas, [39] illustrate the local implementation of the Varna system around 1 Kya, following the arrival of Brahmins into the region [15], [17]. The Tamil epics of this period, such as the Purananuru anthology and Silapathikaram, describe a society with a well-defined occupational class structure based on subsistence practices [22]. Earlier genetic studies of TN populations identified clear differentiations of endogamous ethnic groups classified into Major Population Groups (MPG) based on socio-cultural characteristics reflecting subsistence, traditional occupation, and native language (mother tongue) [40], [41]. Although some studies have identified hill tribes as the earliest settlers, and others suggested a common genetic signature among distantly ranked-caste populations, the main evolutionary and demographic processes shaping the observed genetic differences among populations from TN are still unresolved in the literature [15], [42], [43], [44].

In the present study, we examined the Y-chromosomal lineages of 1,680 individuals sampled from 12 tribal and 19 non-tribal well-defined endogamous populations. We first investigated whether tribal and non-tribal groups shared a common genetic heritage and characterized the proportion of putatively autochthonous and non-autochthonous Indian Y-chromosomal haplogroups. It is important to note that the total sample size used here is higher than those in other studies covering the entire Indian subcontinent. Further, the detailed anthropological annotation of endogamous populations sampled from a restricted region within India, together with the paleoclimatic, archeological and historical regional-background were all important aspects needed to reduce the confounding relationships among socio-cultural factors. This general approach allowed us to infer important genetic signals and the finer details of the population demographic histories. Therefore, we sought to determine which of the classifications based either on the Varna system (rank status, tribe-caste dichotomy), or social-cultural factors (reflecting subsistence, traditional customs and native language), or geography better indicated true endogamous groups by exhibiting higher between-population differences and lower within-population variation. Since both Y chromosome and caste designation are paternally inherited, we further explored whether any of these genetic differences could be attributed to the historical evidences of the establishment of the Hindu Varna system. In contrast, we found the overall Y-chromosomal patterns, the time depth of population diversifications and the period of differentiation correlated better with archeological evidences and the demographic processes of Neolithic agricultural expansions into the region.

## Materials and Methods

### Sampling Strategy

**Figure 1:**
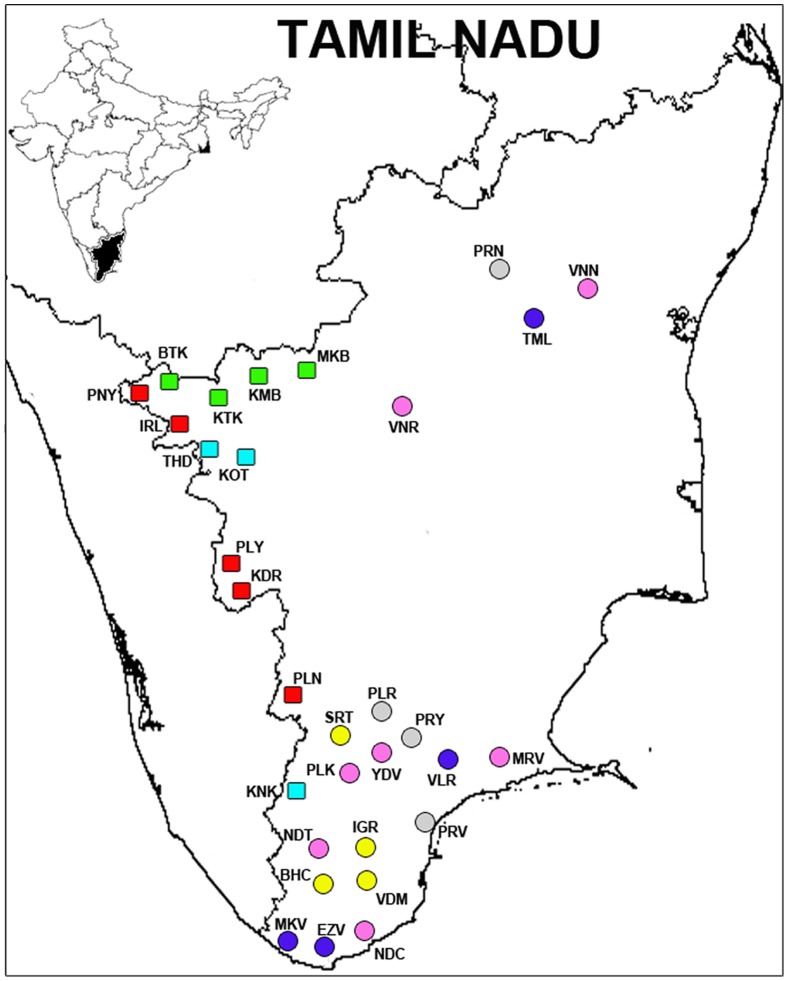
Tamil Nadu map showing the sampling location of the 12 tribal (squares) and 19 non-tribal (circles) populations. The majority of tribal populations are located in the mountains of the Western Ghats. The color codes are: Red – Hill Tribe Foragers (HTF); Turquoise – Hill Tribe Cremating (HTC); Green – Hill Tribe Kannada (HTK); Grey – Schedule Castes (SC); Pink – Dry-Land Farmers (DLF); Deep Blue – Artisan and Warriors (AW) and Yellow – Brahmin related (BRH). Population abbreviations are as shown in Table 1.

Tamil Nadu, the land of Tamils (Tamil has the most ancient literary tradition of all Dravidian languages), is the southeastern most province of India, measuring 130,058 km^2^ with a population of 62,405,679 (2001 Indian Census: http://censusindia.gov.in), the majority living in 17,272 villages. We sampled a total of 1,680 men, avoiding relatives to the third degree, from 12 tribal and 19 non-tribal endogamous populations, which were selected for their cultural uniqueness, geographical spread, and ethnographic features. Samples from tribal participants were collected in their isolated native villages and settlements from the tropical forests of Western Ghats on the west side of TN. In contrast, non-tribal populations exhibit a larger census sizes and geographical spread and they were sampled in colleges and community gatherings, covering 8% of the total villages from TN (see Figure 1 for sampling locations). The institutional Ethical Committees of Madurai Kamaraj University and the University of Pennsylvania (USA) approved the protocol and ethical clearance of the study. The project was explained to the volunteers through local contacts or community leaders in their local languages and signed informed consent was obtained before samples were collected. Permission to utilize pre-existing samples from Nilgiri tribes (N = 570) was obtained from the relevant institution (Nilgiris Adivasi Welfare Association). Further genotyping of 17 Y-STRs and deeper Y-SNPs were performed on 46 samples of Piramalai Kallar, 40 samples of Sourashtra and 107 samples of Yadhava used in a previous study [19].

While many previous Indian population studies aimed to elucidate the main processes involved in the genesis of the social stratification by pooling populations into broad classifications such as caste-tribe dichotomy and social hierarchy [6], [13], [45], [46], we sought to explore whether alternative classifications could better reflect the relationships among the true endogamous groups by increasing between-population differences and reducing within-population variation [24]. We considered a partition of the 31 endogamous populations into seven Major Population Groups (MPG) based on socio-cultural factors primarily reflecting subsistence, traditional customs and native language [47], [48], [49], [50], which we contrasted with alternative groupings. The defining features for these MPGs were the following: (1) ‘Hill Tribe – Foragers’ (HTF), tribal populations sharing a foraging mode of subsistence and speaking their own Dravidian (Tamil/Malayalam) dialects; (2) ‘Hill Tribes – Cremating’ (HTC), tribes who cremate their dead, an unique socio-cultural feature among these tribal populations; (3) ‘Hill Tribes - Kannada-Speakers’ (HTK), hunter-gatherer tribes speaking the Kannada (Dravidian) languages; (4) ‘Scheduled Castes’, (SC), designated by the Indian Government as non-land owning laborers, ranked lowest in the Varna system; (5) ‘Dry Land Farmers’ (DLF), populations living by dry-land farming subsistence, cultivating crops (millets and grains) that do not require irrigation technology; (6) ‘Artisans and Warriors’ (AW), populations that are traditionally warriors or artisans of various kinds, and; (7) ‘Brahmin Related’ (BRH), following the Vedic traditions with a good knowledge on water management and wet land irrigation. The populations included in each of the seven MPG and their ethnographic notes are given in Table 1. Although it may appear that the proxies used for grouping the populations mix criteria in non-uniform and arbitrary ways, we followed a systematic, step-by-step approach to test and validate these classifications by comparing them with other groupings employed in the literature. Endogamous populations were initially sampled taking caste-tribe and social hierarchy into consideration. After considering their ethnographic histories in greater detail, we tested whether tribes with common cultural features tended to share a similar genetic makeup, and whether population groups differentiated better when clustered according to socio-cultural factors reflecting their mode of subsistence, traditional customs, and native language. It is important to stress that many of the criteria used in the classification based on the seven MPG are in some degree correlated with previous methods employed to classify Indian populations (such as tribe-caste dichotomy, or caste-rank hierarchy). It could be argued that the seven MPG method may not be the best possible arrangement from the perspective of explaining the entire cultural variation in TN. However it captures the observed pattern of genetic variation slightly better than any of the previously attempted models (see Results Section). Finally, we recognized that there is always a degree of arbitrary in all the methods used to classify endogamous populations, but all of them are just subtle variations around the same theme: economic or mode of subsistence.

**Table 1:** Description of the 31 tribal and non-tribal endogamous populations studied.

Major Group	Code^h^	Population Name	Linguistic Family	Native Language	Social Rank^j^	Mode of Subsistence	Code^h^	Sampled District	Coordinates^k^ (latitude/longitude)	#	Census
HTF-Hill Tribe Foragers	PNY	Paniya	DR	Tamil/Malayalam	Tribe	Foragers/Cultivators	PNY	Nilgiris	10.6055 ; 77.4056	72	9121^a^
	PLN	Paliyan	DR	Tamil	Tribe	Honey Gatherers	PLN	Theni	9.671 ; 77.2472	95	3,052^a^
	PLY	Pulayar	DR	Tamil/Malayalam	Tribe	Foragers	PLY	Coimbatore	10.3514 ; 76.9068	63	8,406^a^
	IRL	Irula	DR	Tamil	Tribe	Foragers	IRL	Nilgiris	10.6138 ; 77.4056	80	155,606^a^
	KDR	Kadar	DR	Tamil	Tribe	Foragers	KDR	Coimbatore	10.2808 ; 76.9639	28	568^a^
HTC-Hill Tribe Cremating	KNK	Kanikaran	DR	Malayalam	Tribe	Foragers/Shifting Cultivation	KNK	Tirunelveli	9.0952 ; 77.3203	17	3,136^a^
	THD	Thoda	DR	Toda	Tribe	Domestication	THD	Nilgiris	11.1721 ; 77.029	26	1,560^a^
	KOT	Kota	DR	Tamil	Tribe	Domestication/Metallurgy	KOT	Nilgiris	11.1469 ; 76.9713	62	1,140^a^
HTK-Hill Tribe Kannada	BTK	Betta Kurumba	DR	Kannada	Tribe	Honey Gatherers	BTK	Nilgiris	11.6623 ; 76.5278	17	34,747^b^
	KTK	Kattunaickan	DR	Kannada	Tribe	Foragers	KTK	Nilgiris	11.6124 ; 76.9349	46	45,227^a^
	KMB	Kurumba	DR	Kannada	Tribe	Honey Gatherers	KMB	Nilgiris	11.7766 ; 76.9754	35	5,498^a^
	MKB	Mullukurumba	DR	Kannada	Tribe	Foragers	MKB	Nilgiris	11.7081 ; 77.1066	29	4,354^b^
SC-Schedule Caste	PRN	Parayar NTN	DR	Tamil	Low	Agriculture Labourers	PRN	N.Arcot	12.4194 ; 79.1179	52	1,860,519^a^
	PRY	Parayar	DR	Tamil	Low	Agriculture Labourers	PRY	Madurai	9.9392 ; 78.2544	24	1,117,197^c^
	PLR	Pallar	DR	Tamil	Low	Agriculture Labourers	PLR	Tirunelveli	10.0183 ; 78.0292	51	2,272,265^a^
	PRV	Paravar	DR	Tamil	Low	Coastal Fishermen	PRV	Trichendur	8.9904 ; 78.1978	27	2,035^d^
DLF-Dry Land Farmers	YDV	Yadhava	DR	Tamil	Middle	DLF/Cattle keepers	YDV	Madurai	9.8705 ; 78.1316	107	760,041^c^
	VNR	Vanniyar	DR	Tamil	Middle	DLF	VNR	Erode	12.187 ; 78.837	21	760,041^c^^,^ g
	VNN	Vanniyar NTN	DR	Tamil	Middle	DLF	VNN	N.Arcot	12.3596 ; 79.2876	96	760,041^c^^,^ g
	NDT	Nadar TNV	DR	Tamil	Middle	DLF/Toddy Tapping	NDT	Tirunelveli	8.7659 ; 77.4824	59	603,189^e^^,^ g
	NDC	Nadar Cape	DR	Tamil	Middle	DLF/Toddy Tapping	NDC	Kanyakumari	8.1717 ; 77.6037	98	603,189^e^^,^ g
	PLK	Piramalai Kallar	DR	Tamil	Middle	DLF	PLK	Madurai	9.6733 ; 77.7706	53	260,000^b^
	MRV	Maravar	DR	Tamil	Middle	DLF	MRV	Ramnad	9.3365 ; 78.8015	80	423,012^c^
AW-Artisan&Warriors	VLR	Valayar	DR	Tamil	Low	Net Weavers/Hunter Gatherers	VLR	Madurai	9.7465 ; 78.335	95	300,000^d^
	TML	Tamil Jains	DR	Tamil	Middle	Weavers of Mats/Wet Land Agriculture	TML	N.Arcot	12.1719 ; 79.0377	100	100,000^d^
	EZV	Ezhava	DR	Tamil	Middle	Warriors/Toddy Tapping	EZV	Kanyakumari	8.1554 ; 77.4322	95	300,000^d^
	MKV	Mukkuvar	DR	Tamil	Low	Fishnet Weaving/Fishing	MKV	Kanyakumari	8.2144 ; 77.2772	17	100,000^d^
BRH-Brahmins	SRT	Sourashtra	IE	Saurashtri	Middle	Wet Land Agriculture/Weavers	SRT	Madurai	9.8777 ; 77.9301	40	87,149^d^
	BHC	Brahacharanam	IE	Sanskrit^i^	High	Wet Land Agriculture/Priests	BHC	Tirunelveli	8.525 ; 77.4361	21	494,721^c^^,^ f ^,^ g
	IGR	Iyengar	IE	Sanskrit^i^	High	Wet Land Agriculture/Priests	IGR	Madurai	8.6117 ; 77.6522	11	494,721^c^^,^ f ^,^ g
	VDM	Vadama	IE	Sanskrit^i^	High	Wet Land Agriculture/Priests	VDM	Tirunelveli	8.5854 ; 77.7261	63	494,721^c^^,^ f ^,^ g

a - 2001 Census, Government of India, http://censusindia.gov.in/.

b -1981 Indian Census.

c -1931 Indian Census.

d - Estimated census size.

e -1901 Indian Census.

f - All Brahmin-related castes in Tamil Nadu,

g -No information available.

h -Population code used in PCA & MDS plots,

i -Sanskrit is the language of scriptures and ceremonies, but populations quickly adopted local cultures and languages.

j -Lower, Middle & Higher social ranks are self-perceived/assigned classifications.

k -Approximate coordinates.

NTN (North Tamil Nadu),TNV (Tirunelveli).

DR (Dravidian), IE (Indo-European).

### Y-Chromosomal Analysis

DNAs were extracted from blood or mouth-wash samples using standard methods [19]. Samples were genotyped for single nucleotide polymorphisms (SNPs) with a set of 23 custom TaqMan assays (Applied Biosystems) using a 7900HT Fast Real-Time PCR System. In addition, 19 Y-chromosomal short tandem repeat (STR) and 6 SNP loci (Y-filer™ and Multiplex II Kits, ABI) were genotyped using an ABI 3130XL Gene Analyzer, and fragment sizes were determined using the GeneMapper Analysis Software (v3.2, ABI) as described elsewhere [51]. Genotypes were validated by testing reference samples from Coriell and the Genographic Consortium. The multi-copy markers DYS385a and DYS385b were excluded from further analyses because of ambiguity in distinguishing these loci. Y chromosome haplogroups (HGs) and paragroups were determined according to the 2008 YCC nomenclature [52].

### Statistical Analysis

The software ARLEQUIN 3.11 [53] was employed to compute Nei's D (Nei 1987) and conduct AMOVA [54] using both Y-chromosome HG frequencies and haplotype data. Fisher exact tests were carried out among populations and MPGs to identify significantly over- or under-represented HGs. Among those over-represented HGs that tended to characterize any given MPG, Fisher exact tests were further performed on the number of populations over-represented in the given HG within the MPG versus those outside of the MPG to quantify the significance of such associations. Principal Component Analysis (PCA) [55] was performed using HG frequencies, centered without variance normalization [56] and with the significant components identified by employing the skree-plot method [57] using R, version 2.9.1 (http://www.r-project.org/). The same software was implemented to perform non-metric multidimensional scaling (MDS) [58] using R*_ST_* distances generated from the 17 Y-STR data of the TN populations, using ARLEQUIN. The relative HG age estimates were based on the variance of 17 STRs of the most frequent HGs for the seven MPG as previously described [51].

We considered the problem of how to quantify the significance of the difference between specific population group structures. AMOVA's resampling scheme compares individual group structures to the whole ensemble of randomly varied assignments of populations to groups, as well as of samples to populations. This tests the hypothesis that a specific group structure represents organization of the genetics among populations better than would be expected by chance. In our case, we had the different problem of testing whether one group structure was significantly better than another group structure. In this case, assignments were already determined, and likely are both already better than expected by chance. The question we tested was whether that variation in data randomly drawn from a population could have produced sufficient variation in the AMOVA results to account for the differences between the specific group assignments being compared by chance? Hence we resampled the STR haplotypes with replacement, modeled by a multinomial distribution, and computed the median and 95%CI's of the results using R, version 2.9.1. We tested resampling sizes up to 5,000 times, and found that 500 were sufficient to give reasonable accuracy on the median and confidence interval estimates. We therefore resampled each configuration only 500 times.

The phylogenetic relationships among Y-STR haplotypes drawn from individual haplogroups were estimated with the reduced-median (RM) network algorithm in the program Network 4.5.0 [59], [60], applying weights inverse to averaged haplotype variance and reduced median reduction coefficient set at 1.0. This program creates a tree topology based on the interrelationships of the emergence and transmission of mutations in the respective haplotypes. Even under simplifying conditions, the construction of this simple combinatorial structure is algorithmically difficult, and diverse algorithms give different answers. This result can be informative if some subset of the results is consistent among models. Therefore, in addition to using Network for assessing the phylogenetic relationships of Y-STR haplotypes, we also used ULTRANET (http://www.dei.unipd.it/~ciompin/main/Sito/Ultranet.html), where the underlying distance (metric) between nodes is ultrametric. Since tree structures reflect an ultrametric structure, an algorithm that maps the compatibility of associations according to such a structure may be uniquely informative. This approach, which is orthogonal to other phylogenetic approaches, helped confirm the results observed in RM network analysis, thereby validating the consistency of the population associations with evolutionarily related haplotypes.

Coalescence methods, as implemented in BATWING [61], were applied to several different subsets of populations to quantify major underlying demographic events, estimate divergence times and assess the phylogenetic relationships among TN populations. One of the major characteristics of BATWING is that the trees it produces are constructed on the assumption of no gene flow among demes. The proportions of samples the Metropolis-Hastings algorithm provides in each tree gives some sense of the strength of that candidate tree in representing the data. These estimates account for the impact of mutation histories through the likelihood scores obtained over the distributions of priors for mutation rates and other demographic parameters. The outcome of these estimates is that modal, and near modal, trees will show a somewhat filtered view of the genetics contributing to the most likely trees observed. Given these considerations, BATWING is expected a priori to be appropriate for testing whether major population differentiation occurred before or after the Varna system was historically established in TN, under the assumption of restricted admixture among populations under this social organization and structured endogamous system. The various testing procedures described above, including MDS, PCA, the AMOVA tests for differentiation, and the Fisher tests, were further applied to establish whether there was a signal for common gene pools among populations, as required for typical BATWING analyses.

In addition, BATWING admixture validation tests [62] of the TN data were applied under three simulated potential scenarios. In the first scenario, an individual population (Paniya) was randomly split, and the BATWING analysis of the population split time was performed. BATWING generally produced a median time of less than 500 years, with the 95% confidence intervals (CI) covering only the last two generations. In the second scenario, recent gene flow was modeled between two populations (Paniya and Brahacharanam) estimated by BATWING to have already been isolated for a significant time (19.5 Kya) by randomly mixing different proportions of chromosomes from each population. BATWING gave much younger population divergence estimates (9.3 Kya) than the unmixed split, even with only 5% of the Y-chromosomes mixed randomly between the two populations, with a 10% mix between populations decreased the divergence time estimates by more than 50% (3 Kya). In the third scenario, we explored the impact of BATWING estimates by randomly introducing an in-migrating population (Paniya) carrying new paternal lineages into two differentiated demes (Brahacharanam and Kota: split time was estimated at 4.7 Kya). These estimates were only slightly affected (the split time actually appeared to increase to 6.2 Kya) when the in-migrating proportion did not exceed more than 40–50%. At that point, the modal trees were dominated by the in-migrating population. Overall, the results of the BATWING admixture tests based on data from the TN populations were similar to those observed in a study of religious populations within Lebanon [62]. Therefore, BATWING generally seems to show little sensitivity to gene flow from immigrants bringing new paternal lineages (different HGs) into the parent population, but is very sensitive to gene flow between populations sharing paternal lineages from the same HGs.

Besides assuming no gene flow, BATWING presupposes that the population samples are random. As a result, using BATWING to analyze the histories of individual HGs drawn from populations yields dramatically different estimates of coalescence times, times of expansion, and other population parameters because, as mentioned in the admixture modeling, BATWING is more sensitive to admixture than in-migration. Thus, BATWING may be applied to individual HGs to extract information about specific in-migration events. Further, HGs that tend to correlate strongly with overall population estimates are likely to be more representative of their common ancestral gene pool. These results may be expected in that selection of the modal population trees will tend to preserve configurations where the most common of the shared lineages comprise the strongest signals contributing to the likelihood function. Therefore, selection of modal trees acts as a filter that tends to exclude immigrating contributions, although it will be heavily influenced by inter-population migration.

In these BATWING estimates, mutation rate priors were those previously proposed [63] based on the effective mutation rates previously cited [64]. Between 1.5 and 3.5 million Monte Carlo (MC) samples were collected, generally accepting equilibration following 500,000 MC samples and being determined by decay to equilibrium of global estimates of effective population size and relative constancy of quantile measurements extracted from the equilibrated regions. Times associated with clusters identified by RM networks as indicating evolution within populations were estimated using UEPtmin and UEPtmax estimates within BATWING. When computing population splits, large numbers of populations tend to produce cross-talk between bifurcations on different branches. A way to resolve this cross-talk is to set up multiple runs with the various branches pooled except for the primary branch under consideration. This approach also provides an opportunity to check the consistency of split times of the parent branches common to the pooled topologies. Composite trees may then be constructed from the results of the multiple runs. SNPs selected as unique evolutionary polymorphisms (UEPs) in computations of population split times depended on the representation of variation through each of the populations being considered, or through the pooled populations for UEP time estimates.

## Results

### NRY landscape of Tamil Nadu reveals predominantly autochthonous lineages

A total of 21 Y chromosome HGs were identified in the study populations (Table 2). The overall HG diversity among populations was 0.886±0.003; of these, tribal populations exhibited lower diversity (0.796±0.013) than non-tribal populations (0.881±0.004). The majority of this genetic variation (82%) was accounted for by seven HGs: H1-M52 (17.4%), F*-M89 (16.3%), L1-M27 (14.0%), R1a1-M17 (12.7%), J2-M172 (9.4%), R2-M124 (8.2%) and H-M69 (4.7%). It should be noted that 90% of the C-M130 samples reported here (66 out of 74) were positive for C5-M356 while the rest were negative for both C3-M217 and C5-M356 (Table S1).

**Table 2:** Y chromosome haplogroup frequencies (%) in the 31 populations from Tamil Nadu.

POPULATIONS		N	C-M130	E-M96	F-M89	G-M201	H-M69	H1-M52	H1a-M197	H2-Apt	J-M304	J2-M172	J2a1-M47	J2a3-M68	K-M9	L1-M27	L3-M357	O-M175	P-M45	Q-M242	R-M207	R1a1-M17	R2-M124	Nei Gene Diversity (SD)
**HTF-Hill Tribe Foragers**																								
Paniya		72	15.28	0.00	75.00	0.00	0.00	0.00	0.00	0.00	0.00	0.00	0.00	1.39	1.39	0.00	0.00	0.00	1.39	1.39	2.78	1.39	0.418 (0.067)	0.00
Paliyan		95	10.53	0.00	55.79	2.11	2.11	11.58	0.00	0.00	0.00	0.00	0.00	0.00	2.11	3.16	0.00	0.00	0.00	0.00	3.16	0.00	0.659 (0.049)	9.47
Pulayar		63	1.59	0.00	57.14	0.00	6.35	11.11	0.00	0.00	0.00	0.00	0.00	0.00	1.59	1.59	0.00	1.59	3.17	0.00	0.00	0.00	0.640 (0.060)	15.87
Irula		80	6.25	0.00	36.25	0.00	18.75	7.50	0.00	8.75	0.00	0.00	0.00	0.00	16.25	0.00	0.00	0.00	1.25	1.25	0.00	2.50	0.799 (0.028)	1.25
Kadar		28	10.71	0.00	28.57	0.00	0.00	32.14	0.00	0.00	0.00	0.00	0.00	0.00	0.00	0.00	0.00	0.00	0.00	0.00	0.00	0.00	0.749 (0.032)	28.57
	***HTF Total***	***338***	***8.88***	***0.00***	***53.25***	***0.59***	***6.21***	***9.76***	***0.00***	***2.07***	***0.00***	***0.00***	***0.00***	***0.30***	***5.03***	***1.18***	***0.00***	***0.30***	***1.18***	***0.59***	***1.48***	***0.89***	***0.687 (0.025)***	***8.28***
**HTC-Hill Tribe Cremating**																								
Kanikaran		17	0.00	0.00	11.76	5.88	0.00	29.41	0.00	0.00	0.00	0.00	0.00	0.00	23.53	0.00	0.00	0.00	5.88	5.88	5.88	5.88	0.875 (0.058)	5.88
Thoda		26	7.69	0.00	3.85	0.00	0.00	11.54	0.00	0.00	0.00	0.00	38.46	0.00	7.69	3.85	3.85	3.85	0.00	0.00	0.00	11.54	0.834 (0.061)	7.69
Kota		62	0.00	0.00	8.06	0.00	1.61	30.65	0.00	0.00	0.00	0.00	6.45	1.61	0.00	0.00	0.00	0.00	4.84	4.84	22.58	19.35	0.815 (0.026)	0.00
	***HTC Total***	***105***	***1.9***	***0.00***	***7.62***	***0.95***	***0.95***	***25.71***	***0.00***	***0.00***	***0.00***	***0.00***	***13.33***	***0.95***	***5.71***	***0.95***	***0.95***	***0.95***	***3.81***	***3.81***	***14.29***	***15.24***	***0.867 (0.016)***	***2.86***
**HTK-Hill Tribe Kannada**																								
Betta Kurumba		17	0.00	0.00	58.82	0.00	0.00	11.76	0.00	0.00	0.00	0.00	0.00	0.00	17.65	0.00	0.00	0.00	0.00	0.00	5.88	5.88	0.640 (0.116)	0.00
Kattunaickan		46	2.17	0.00	21.74	0.00	17.39	41.3	0.00	0.00	2.17	0.00	0.00	0.00	0.00	0.00	0.00	0.00	2.17	4.35	0.00	4.35	0.761 (0.044)	4.35
Kurumba		35	2.86	0.00	11.43	0.00	2.86	65.71	0.00	0.00	0.00	0.00	0.00	2.86	5.71	0.00	0.00	0.00	0.00	0.00	2.86	5.71	0.561 (0.096)	0.00
Mullukurumba		29	0.00	0.00	20.69	0.00	0.00	34.48	0.00	0.00	0.00	0.00	0.00	3.45	24.14	0.00	0.00	0.00	0.00	0.00	0.00	17.24	0.776 (0.036)	0.00
	***HTK Total***	***127***	***1.57***	***0.00***	***23.62***	***0.00***	***7.09***	***42.52***	***0.00***	***0.00***	***0.79***	***0.00***	***0.00***	***1.57***	***9.45***	***0.00***	***0.00***	***0.00***	***0.79***	***1.57***	***1.57***	***7.87***	***0.748 (0.028)***	***1.57***
**SC-Schedule Caste**																								
Parayar NTN		52	7.69	0.00	3.85	1.92	3.85	34.62	0.00	0.00	0.00	0.00	0.00	0.00	9.62	1.92	1.92	0.00	1.92	1.92	3.85	9.62	0.836 (0.037)	17.31
Parayar		24	4.17	0.00	0.00	8.33	0.00	20.83	0.00	0.00	4.17	0.00	0.00	0.00	12.50	4.17	4.17	0.00	0.00	8.33	12.50	8.33	0.920 (0.029)	12.50
Pallar		51	1.96	0.00	5.88	7.84	5.88	11.76	0.00	1.96	0.00	0.00	1.96	0.00	15.69	5.88	0.00	0.00	1.96	1.96	13.73	9.80	0.914 (0.015)	13.73
Paravar		27	0.00	0.00	3.70	0.00	0.00	14.81	0.00	0.00	0.00	0.00	0.00	0.00	18.52	0.00	7.41	0.00	0.00	3.70	3.70	11.11	0.815 (0.052)	37.04
	***SC Total***	***154***	***3.9***	***0.00***	***3.90***	***4.55***	***3.25***	***21.43***	***0.00***	***0.65***	***0.65***	***0.00***	***0.65***	***0.00***	***13.64***	***3.25***	***2.60***	***0.00***	***1.30***	***3.25***	***8.44***	***9.74***	***0.880 (0.012)***	***18.83***
**DLF-Dry Land Farmers**																								
Yadhava		107	2.80	0.00	5.61	1.87	3.74	19.63	0.00	0.00	0.00	0.00	0.00	1.87	20.56	0.00	0.93	0.00	0.00	0.93	14.95	10.28	0.860 (0.013)	16.82
Vanniyar		21	0.00	0.00	9.52	4.76	0.00	4.76	0.00	0.00	0.00	0.00	9.52	0.00	28.57	0.00	0.00	0.00	0.00	0.00	14.29	14.29	0.876 (0.043)	14.29
Vanniyar NTN		96	7.29	1.04	8.33	3.13	3.13	13.54	0.00	3.13	0.00	0.00	2.08	0.00	23.96	2.08	2.08	0.00	0.00	2.08	11.46	9.38	0.889 (0.016)	7.29
Nadar TNV		59	0.00	0.00	8.47	8.47	11.86	15.25	0.00	1.69	0.00	0.00	0.00	0.00	28.81	0.00	0.00	0.00	3.39	0.00	6.78	10.17	0.861 (0.025)	5.08
Nadar Cape		98	4.08	4.08	5.10	9.18	7.14	7.14	0.00	1.02	0.00	0.00	1.02	0.00	23.47	0.00	1.02	1.02	9.18	1.02	12.24	4.08	0.895 (0.015)	9.18
Piramalai Kallar		53	9.43	0.00	5.66	3.77	3.77	16.98	0.00	1.89	0.00	0.00	0.00	1.89	47.17	1.89	0.00	0.00	0.00	0.00	1.89	3.77	0.745 (0.055)	1.89
Maravar		80	0.00	0.00	3.75	8.75	5.00	10.00	1.25	1.25	0.00	0.00	0.00	3.75	10.00	0.00	1.25	0.00	2.50	7.50	16.25	15.00	0.904 (0.011)	13.75
	***DLF Total***	***514***	***3.70***	***0.97***	***6.23***	***5.64***	***5.25***	***13.23***	***0.19***	***1.36***	***0.00***	***0.00***	***0.97***	***1.17***	***24.12***	***0.58***	***0.97***	***0.19***	***2.53***	***1.95***	***11.67***	***9.14***	***0.881 (0.007)***	***10.12***
**AW-Artisan Warriors**																								
Valayar		95	6.32	0.00	12.63	2.11	8.42	10.53	0.00	1.05	0.00	0.00	0.00	0.00	8.42	2.11	0.00	2.11	1.05	1.05	20	15.79	0.890 (0.012)	8.42
Tamil Jains		100	4.00	0.00	2.00	2.00	3.00	22.00	0.00	3.00	0.00	0.00	0.00	1.00	9.00	2.00	2.00	0.00	1.00	0.00	18.00	20.00	0.862 (0.015)	11.00
Ezhava		95	0.00	0.00	2.11	3.16	5.26	25.26	0.00	0.00	0.00	1.05	0.00	0.00	20.00	1.05	0.00	0.00	0.00	0.00	24.21	5.26	0.823 (0.017)	12.63
Mukkuvar		17	0.00	0.00	0.00	0.00	0.00	17.65	0.00	11.76	0.00	0.00	0.00	0.00	5.88	0.00	0.00	0.00	0.00	11.76	11.76	23.53	0.890 (0.040)	17.65
	***AW Total***	***307***	***3.26***	***0.00***	***5.21***	***2.28***	***5.21***	***19.22***	***0.00***	***1.95***	***0.00***	***0.33***	***0.00***	***0.33***	***12.05***	***1.63***	***0.65***	***0.65***	***0.65***	***0.98***	***20.20***	***14.33***	***0.870 (0.007)***	***11.07***
**BRH-Brahmins**																								
Sourashtra		40	7.50	0.00	0.00	0.00	0.00	25.00	0.00	0.00	0.00	0.00	0.00	0.00	20.00	0.00	0.00	0.00	0.00	0.00	40.00	5.00	0.747 (0.041)	2.50
Brahacharanam		21	0.00	0.00	0.00	0.00	0.00	9.52	0.00	9.52	0.00	0.00	0.00	0.00	4.76	0.00	0.00	4.76	0.00	19.05	33.33	4.76	0.848 (0.054)	14.29
Iyengar		11	0.00	0.00	0.00	27.27	0.00	9.09	0.00	0.00	0.00	0.00	0.00	9.09	0.00	0.00	0.00	0.00	0.00	0.00	36.36	0.00	0.818 (0.083)	18.18
Vadama		63	3.17	0.00	1.59	4.76	0.00	7.94	0.00	3.17	0.00	0.00	0.00	1.59	14.29	1.59	3.17	0.00	0.00	6.35	47.62	0.00	0.746 (0.052)	4.76
	***BRH Total***	***135***	***3.70***	***0.00***	***0.74***	***4.44***	***0.00***	***13.33***	***0.00***	***2.96***	***0.00***	***0.00***	***0.00***	***1.48***	***13.33***	***0.74***	***1.48***	***0.74***	***0.00***	***5.93***	***42.22***	***2.22***	***0.779 (0.030)***	***6.67***
31 populations TOTAL	1680	4.4	0.3	16.25	3.10	4.70	17.38	0.06	1.49	0.12	9.35	0.06	1.19	0.77	13.99	1.13	0.83	0.36	1.55	2.02	12.74	8.21	0.886 (0.003)	

SD (Standard Deviation).

The geographical origins of many of these HGs are still debated. However, the associated high frequencies and haplotype variances of HGs H-M69, F*-M89, R1a1-M17, L1-M27, R2-M124 and C5-M356 within India, have been interpreted as evidence of an autochthonous origins of these lineages during late Pleistocene (10–30 Kya), while the lower frequency within the subcontinent of J2-M172, E-M96, G-M201 and L3-M357 are viewed as reflecting probable gene flow introduced from West Eurasian Holocene migrations in the last 10 Kya [6], [7], [16], [23]. Assuming these geographical origins of the HGs to be the most likely ones, the putatively autochthonous lineages accounted for 81.4±0.95% of the total genetic composition of TN populations in the present study. These results are concordant with earlier studies based on autosomal markers and haploid loci in suggesting lower gene flow from West and Central Asia to south India compared to north India [5], [11], [23], [65]. Additionally, our results indicate a potentially differential genetic impact of these migrations on tribal versus non-tribal groups. For example, the proportion of non-autochthonous Indian lineages was found to be significant higher (*p*<0.0001) among non-tribal populations (13.7±1.03%) than among the tribal populations (7.4±1.09%). In contrast, the proportion of likely autochthonous lineages among the tribal populations (87.7±1.37%) was significant higher (Fisher test: *p*<0.0001) than in non-tribal populations (78.1±1.24%).

### Genetic structure of Tamil Nadu populations is best correlated with subsistence practices

AMOVA using both HGs and STR distances (R*_ST_*) was applied to several different models of population differentiation to assess the proportion of genetic variation explained by geography, tribe-caste dichotomy, caste-rank hierarchy, and other socio-cultural factors reflecting subsistence practices (Table 3, Table S2). The highest genetic variation among classifications involving all populations (*F_CT_* = 0.065; among resampled data, median = 0.064, 95%CI :0.052–0.078) and the lowest variation within groups (*F_SC_* = 0.040; median = 0.062; 0.05–0.074) were observed when populations were classified into the seven MPGs based on subsistence. Further analyses considering only the four non-tribal groups revealed a four-fold decrease in genetic variation among groups (*F_CT_* = 0.015; median = 0.014; 0.003–0.026) when compared to the three tribal groups alone (*F_CT_* = 0.095; median = 0.095; 0.066–0.129). Moreover, the exclusion of HTF reduced the between-group variance by more than two-fold (6.5% to 2.7%), while exclusion of HTK and BRH had little impact. On the other hand, the exclusion of BRH from non-tribal groups reduced the between-group variation threefold (1.5% to 0.4%).

**Table 3:** Analysis of molecular variance (AMOVA).

Populations Grouping	No of groups	Among groups (Fct)		Among populations within groups (Fsc)		Within populations (Fst)	
		SNPs	STRs	SNP^a^	STR^a^	SNP^a^	STR^a^
All 31 populations	1					0.103	0.093
Geography	9	0.025^c^	0.035^b^	0.083	0.063	0.106	0.096
**Socio-Cultural Factors**							
7 Major Populations Groups (MPG)	7	0.082^a^	0.065^a^	0.036	0.040	0.114	0.102
HTF excluded	6	0.035^a^	0.026^a^	0.027	0.034	0.061	0.060
BRH excluded	6	0.077^a^	0.059^a^	0.037	0.042	0.111	0.099
HTK excluded	6	0.082^a^	0.062^a^	0.031	0.039	0.111	0.099
Caste vs Tribe	2	0.075^a^	0.062^b^	0.069	0.065	0.139	0.124
TR-UP-MID-LOW	4	0.057^a^	0.047^a^	0.065	0.063	0.119	0.107
**Tribes Only**							
HTF-HTK-HTC	3	0.110^c^	0.095^a^	0.081	0.079	0.182	0.167
**Non-tribes (Castes) Only**							
UP-MID-LOW	3	0.019^b^	0.015^b^	0.024	0.030	0.042	0.044
SC-DLF-AW-BRH	4	0.023^a^	0.015^b^	0.017	0.026	0.039	0.041
SC -DLF-AW	3	0.009^c^	0.004^d^	0.016	0.027	0.025	0.031

a *P*<0.00001.

b *P*<0.001.

c *P*<0.01.

d No Significant, *P*<0.2.

TR (Tribes), HTF (Hill Tribe Foragers), BRH (Brahmins), HTK (Hill Tribe Kannada speakers), SC (Schedule Castes), DLF (Dry Land Farmers), AW (Artisan & Warriors).

HG, MID, LOW – High, Middle and Low caste-rank hierarchy as described in Table 1.

Endogamous populations were grouped based on geography, tribe-caste dichotomy, caste-rank hierarchy, and socio-cultural features mainly reflecting subsistence (7 Major Population Groups, MPG). The maximal genetic variation among groups (*F_CT_*) and the minimal variation among populations within groups (*F_SC_*) was observed when populations were grouped based on the 7 MPG classification.

To determine if the number of groups taken into consideration had a significant impact on the *F_CT_* values obtained, we compared the mean and 95% CI of the null distribution of Va (among group variance, data not presented) that is used to estimate the *F_CT_* index. It is logical that the Va null distribution would vary with different groupings if the relative impact of groups is high. Contrary to this, we found that the mean and the standard deviations of the null distribution did not vary much among groupings (Table 3) hence suggesting that the number of groups taken in to consideration did not have much impact on the *F_CT_* estimates. Further, the 95% CI intervals of the AMOVA estimates computed by re-sampling 500 haplotypes with replacement across populations showed that 95% CI of 7-MPG classification was significantly higher from that of grouping by geography or Varna rank status (Table S2).

**Figure 2:**
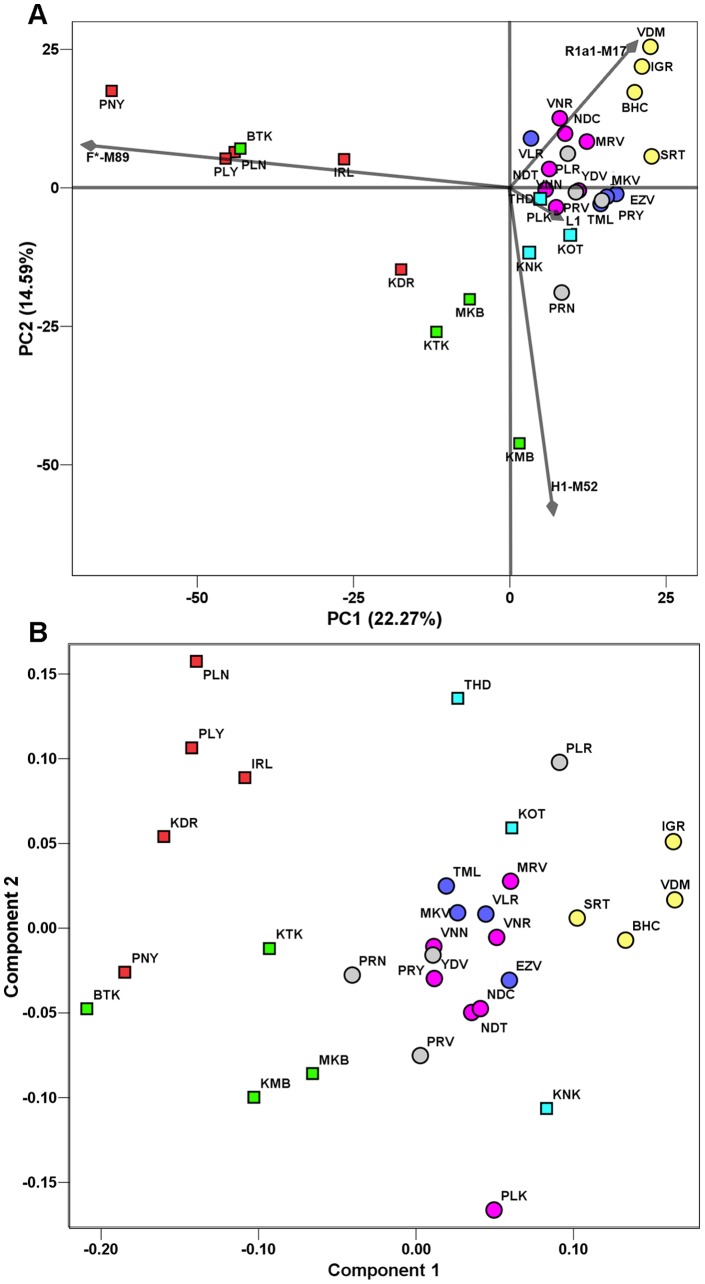
Plots representing the genetic relationships among the 31 tribal and non-tribal populations of Tamil Nadu. (A) PCA plot based on HG frequencies. The two dimensions display 36% of the total variance. The contribution of the first four HGs is superimposed as grey component loading vectors: the HTF populations clustered in the direction of the F-M89 vector, HTK in the H1-M52 vector, BRH in the R1a1-M17 vector, while the HG L1-M27 is less significant in discriminating populations. (B) MDS plot based on 17 microsatellite loci *R_st_* distances. The two tribal groups (HTF and HTK) are clustered at the left side of the plot while BRH form a distant cluster at the opposite side. The colors and symbols are the same as shown in Figure 1, while population abbreviations are as shown in Table 1.

The PCA and MDS analyses of HG frequencies and R*_ST_* distances reflected the AMOVA results (Figures 2a, 2b). In the PCA analysis the first two components accounted for 38.86% variance, while in the MDS analysis a stress value of 15.6% was obtained when the objects were clustered in two dimensions. This stress value is significant in the light of the work of Sturrock and Rocha, 2000 [66]. In both plots, two tribal (HTF, HTK) and the non-tribal Brahmin (BRH) groups formed distinct and distant clusters, while the rest were interspersed in their midst.

Interestingly, the same tribal groups showed greater genetic similarities to other Dravidian tribes from the southern states of Andhra Pradesh and Orissa, and TN BRH clustered with IE speaking populations from multiple regions, when the present data set was compared with 97 populations from India and neighboring regions by PCA (Figure S1, Table S3). The historical migrations of BRH into TN and the long-term isolation for some Dravidian tribal groups already reported in previous studies [15], [17], [25] could potentially explain why HTF, HTK and BRH groups exhibited greater genetic similarities with those culturally related populations outside of TN. Taken together; the PCA, MDS and AMOVA results all indicate strong genetic structure among TN populations. They further suggest that the MPG classification based on socio-cultural factors reflecting subsistence better reproduces true endogamous groups by increasing between-population differences and reducing within-population variation.

### Non-homogenous HG distributions among constituent populations of MPGs

Fisher exact tests indicated that various HGs were significantly predominant in one or another MPG (Table S4). The highest frequency of F-M89 (53.3%) was observed among HTF (p<0.0001), while H1-M52 showed the highest frequency (42.5%) in HTK (p<0.0001). Among the non-tribal groups, BRH showed 42.2% of R1a1-M17 (p<0.0001), and L1-M27 appeared at a higher frequency (24.1%; p<0.0001) among DLF. However, wide variation in HG frequency and composition was observed among the populations included in each of these MPGs (Table 2). For example, the proportion of F*-M89 in HTF ranged from 75% to 28.6% among the constituent populations. A similar pattern was observed in other MPGs characterized by H1-M52 in HTK and L1-M27 in DLF. Thus, not all the constituent endogamous populations in a MPG shared a similar genetic makeup, indicating the differential influence of evolutionary forces such as drift, fragmentation, long-term isolation or admixture.

In addition, Fisher exact tests were used to determine the probability of observing multiple populations within an MPG sharing the same over- or under-represented HGs by chance (e.g., random demic assimilation into a MPG from already differentiated endogamous populations) or because of the systemic inheritance of ancestral lineages among the constituting populations of MPGs. Our results rejected the hypothesis that random processes could have caused the significant over-representation of F*-M89 in HTF+HTK populations (p<0.0001), L1-M27 in DLF populations (p<0.001), H1-M52 in HTK populations (p<0.0001), and R1a1-M17 in BRH populations (*p* = 0.001). Likewise, significant results were obtained for under-representation of F*-M89 in all BRH populations (*p* = 0.043), L1-M27 in HTF populations (*p* = 0.02) and R1a1-M17 in HTF populations (*p* = 0.003). Together, these results argue for the distinctiveness of the ancestral gene pools for MPGs and the shared heritage of these paternal lineages among populations within MPGs, in spite of their non-homogenous distribution. Further, the over-represented HGs marking MPGs explains in part some of the organization observed in the PCA and MDS results, and also yields insight into the differentiations noted in the AMOVA results.

### Reduced median network analysis identifies strong founder effects among tribal populations

RM networks were constructed to evaluate HG diversification within TN populations. Here, low-reticulated networks with branches showing segregation by population were expected if strong founder effects had shaped variation in paternal lineages, particularly in the HGs overrepresented in MPGs. By contrast, reticulated networks exhibiting shared STR haplotypes between populations from different MPGs would indicate that contemporary populations were derived from descendants drawn from differing sources carrying disparate and diverse STR haplotypes, suggesting potential admixture among populations. Long branches with multiple unoccupied steps (internodes) connecting constituent haplotypes would suggest strong genetic drift or possibly sporadic intrusion from a genetically distinct source.

**Figure 3:**
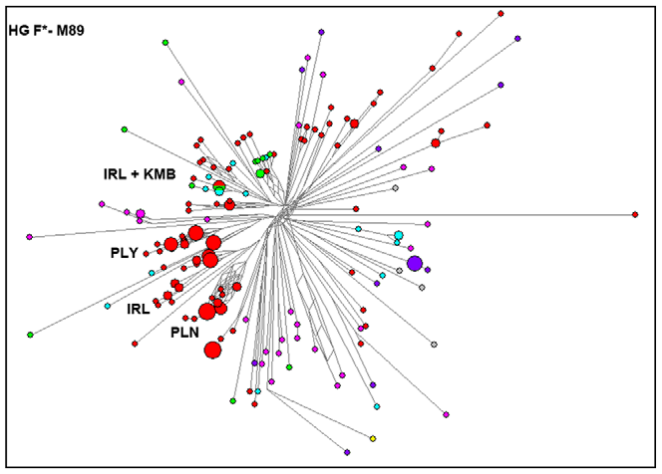
Reduced median network of 17 microsatellite haplotypes within haplogroup F-M89. The network depicts clear isolated evolution among HTF populations with a few shared haplotypes between Kurumba (HTK) and Irula (HTF) populations. Circles are colored based on the 7 Major Population Groups as shown in Figure 1, and the area is proportional to the frequency of the sampled haplotypes. Branch lengths between circles are proportional to the number of mutations separating haplotypes.

F*-M89 was the only HG showing clear population-specific clusters (Paniya, Paliyan and Irula of HTF) suggesting long-term isolation (Figure 3). In contrast, all other RM networks did not show any population-specific clusters and were reticulated with long branches having multiple internodes (Figure S2a to S2e). Overall, these results suggest that both genetic drift (possibly due to founder effects) and admixture may be a common feature of the studied populations. The combination of low segregation among RM networks and higher diversity may result from a period of assimilation of diverse sources into a larger common gene pool from which the modern populations were subsequently drawn.

### HG age estimates are older in non-tribal groups

Tribes are generally considered as the descendants of the early settlers of India and, therefore, better depict the autochthonous genetic composition of India than non-tribal populations [2], [12], [15], [67]. Association between high frequency and high STR variance of a HG in a population are potential indicators of long-term in-situ diversification. These may also indicate the likely source of the HG in other populations. We therefore investigated whether tribal populations possess older genetic lineages, and could thus be the potential sources of these lineages for other populations, by computing HG age estimates based on Y-STR variances (Table 4). The age estimates for all HGs exceeded 10–15 Kya with overlapping confidence intervals among MPGs. Further, MPG exhibiting high frequencies of specific HGs did not show the oldest age estimates. Interestingly, non-tribal groups exhibited older age estimates than tribal groups for all HGs, excepting R2-M124. These results indicated that tribal and non-tribal populations share a genetic heritage dating back to at least the late Pleistocene (10–30 Kya). The HG age estimates presented here are similar to those generated for the same HGs in earlier studies involving a similar or lesser number of samples taken from a broader geographic region of India [7], [23].

**Table 4:** Haplogroup variances and age estimates based on 17 microsatellite loci.

Haplogroup		All MPG	HTF	HTK	HTC	SC	DLF	AW	BRH
**C-M130**	Var (SE)	0.801 (0.176)	0.805 (0.220)			0.682 (0.207)	0.885 (0.181)	0.474 (0.202)	0.394 (0.076)
	Age (SD)	29,029 (6,387)	29,156 (7,987)			24,723 (7,518)	32,057 (6,571)	17,175 (7,308)	14,280 (2,752)
**F-M89**	Var (SE)	0.810 (0.142)	0.687 (0.126)	0.674 (0.136)	0.525 (0.207)	0.704 (0.204)	0.851 (0.194)	0.773 (0.158)	
	Age (SD)	29,345 (5,137)	24,895 (4,560)	24,418 (4,946)	19,017 (7,515)	25,504 (7,410)	30,827 (7,021)	28,026 (5,721)	
**G-M201**	Var (SE)	0.829 (0.182)				0.939 (0.318)	0.536 (0.124)	1.048 (0.317)	0.820 (0.267)
	Age (SD)	30,037 (6,602)				34,009 (11,531)	19,413 (4,495)	37,957 (11,488)	29,696 (9,660)
**H-M69**	Var (SE)	1.327 (0.591)	0.608 (0.226)	0.550 (0.227)		1.456 (0.521)	0.906 (0.376)	1.182 (0.372)	
	Age (SD)	48,073 (21,408)	22,048 (8,177)	20,641 (8,224)		52,749 (18,888)	32,822 (13,629)	42,817 (13,479)	
**H1-M52**	Var (SE)	0.413 (0.078)	0.342 (0.096)	0.294 (0.080)	0.203 (0.039)	0.27 (0.063)	0.508 (0.113)	0.508 (0.108)	0.593 (0.122)
	Age (SD)	14,961 (2,814)	12,390 (3,475)	10,652 (2,905)	7,343 (1,423)	9,782 (2,301)	18411 (4,090)	18,397 (3,921)	21,483 (4,432)
**H2-Apt**	Var (SE)	0.594 (0.106)	0.328 (0.176)				0.441 (0.113)	0.480 (0.226)	0.672 (0.206)
	Age (SD)	21,524 (3,825)	11,874 (6,382)				15,964 (4,107)	17,405 (8,172)	24,332 (7,475)
**J2-M172**	Var (SE)	0.734 (0.101)	0.420 (0.102)			0.717 (0.131)	0.687 (0.119)	0.998 (0.136)	0.762 (0.172)
	Age (SD)	26,598 (3,654)	15,205 (3,706)			25,979 (4,748)	24,898 (4,321)	36,176 (4,946)	27,605 (6,244)
**J2a3-M68**	Var (SE)	0.289 (0.109)			0.266 (0.140)		0.229 (0.114)		
	Age (SD)	10,461 (3,937)			9,629 (5,069)		8,312 (4,119)		
**L1-M27**	Var (SE)	0.414 (0.095)	0.354 (0.124)	0.218 (0.104)	0.309 (0.117)	0,464 (0.13)	0.420 (0.099)	0.416 (0.097)	0.458 (0.132)
	Age (SD)	15,007 (3,460)	12,812 (4,483)	7,890 (3,755)	11,189 (4,242)	16,811 (4,710)	15,236 (3,585)	15,090 (3,531)	16,601 (4,781)
**L3-M357**	Var (SE)	0.220 (0.056)	0.348 (0.153)			0.176 (0.062)		0.182 (0.071)	
	Age (SD)	7,982 (2,021)	12,610 (5,542)			6,394 (2,252)		6,607 (2,585)	
**R-M207**	Var (SE)	0.972 (0.183)			0.730 (0.191)	0.582 (0.126)	1.254 (0.396)		0.985 (0.204)
	Age (SD)	35,203 (6,633)			26,463 (6,921)	21,099 (4,558)	45,444 (14,351)		35,691 (7,382)
**R1a1-M17**	Var (SE)	0.413 (0.060)	0.335 (0.073)		0.387 (0.088)	0.500 (0.135)	0.456 (0.074)	0.365 (0.047)	0.369 (0.062)
	Age (SD)	14,974 (2,169)	12,148 (2,653)		14,006 (3,200)	18,124 (4,878)	16,510 (2,684)	13,229 (1,721)	13,387 (2,261)
**R2-M124**	Var (SE)	0.652 (0.111)		1.048 (0.237)	0.328 (0.137)	0.584 (0.121)	0.597 (0.115)	0.642 (0.171)	
	Age (SD)	23,638 (4,023)		37,960 (8,588)	11,880 (4,975	21,164 (4,401)	21,622 (4,182)	23,246 (6,211)	

Var (Variance), SE (Standard Error), SD (Standard Deviation).

Haplogroup age estimates are given in years; groups with less than 5 STRs (samples) were excluded from calculations. Non-tribal groups (castes) displayed the oldest age estimates for most of the Y chromosome haplogroups.

### BATWING estimates of genetic affinity and ancestry

**Figure 4:**
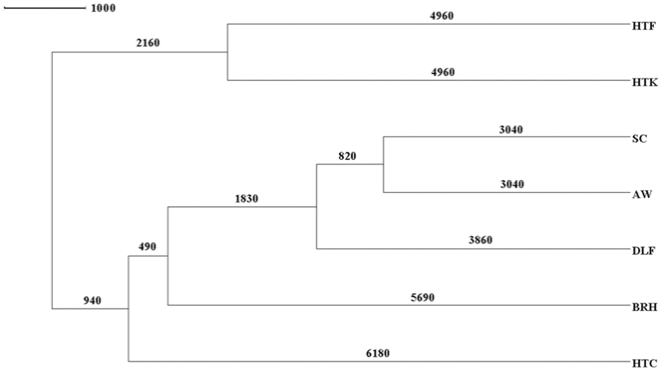
Modal tree obtained by BATWING indicating the coalescence time divergence estimates (in years) among Major Populations Groups (MPG) after using 17 STRs from all haplogroups. BATWING estimates suggest that all populations groups started to diverge 7.1 Kya (95% CI: 5.5–9.2 Kya), with limited admixture among them for the last 3.0 Kya (2.3–4.3 Kya), the youngest diverge time estimate. The modal tree shows two differentiated nodes with clear overlapping estimates of the splits: a first node including one of the tribal groups (HTC) together with all the non-tribal MPGs (castes) with a divergence time of 6.2 Kya (4.7–8.0 Kya), while the second node embraces the HTF and HTK tribal groups with an estimated divergence between then of 4.9 Kya (3.6–7.1 Kya).

We configured several BATWING runs using different subsets of data to estimate the dates of population differentiation and explore the different demographic processes and affinities among the MPGs and their constituent populations. The first set of BATWING runs analyzed haplotypes from all HGs among all of the MPGs to investigate whether tribal and non-tribal MPGs have an independent origin or instead descended from a common ancestral gene pool. If tribal and non-tribal groups have independent origins, then it would be expected that population tree bifurcations marking the differentiation of these two groupings would exhibit very old divergence time estimates and non-overlapping confidence intervals (CIs). Figure 4 represents the modal tree obtained for this BATWING run. It shows that populations begin to diverge around 7.1 Kya (95% CI: 5.5–9.2 Kya), and contains two differentiated nodes with clear overlapping estimates of the splits. The first node separated the HTF and HTK tribal groups from the rest of the MPGs, with an estimated divergence time of 4.9 Kya (3.6–7.1 Kya), while the second included the other tribal group (HTC) and the non-tribal MPGs, with a divergence time of 6.2 Kya (4.7–8.0 Kya). These BATWING estimates suggest that all MPGs started to diverge during the same span of time with very limited admixture among them, at least for the last 3 Kya (2.3–4.3 Kya), the youngest time estimate.

The second set of BATWING runs included only haplotypes from one of the most common HGs among MPGs. In this regard, we would like to emphasize that BATWING results using haplotypes from only one HG cannot be interpreted as population divergence times, but rather reflect the demographic histories of the specific paternal lineage among populations. Also, deviations from population estimates among the different runs could reflect in-migrations (gene flow) involving a particular HG rather than multiple paternal lineages obtained from assimilation from a common ancestral gene pool. For these reasons, we explored whether the paternal lineages for each HG originated from the MPG that exhibits the highest frequency of this HG as a way to identify sources and recipients of these Y-chromosomes. In addition, similar splitting patterns obtained for the different HG trees could be interpreted as demonstrating that the paternal lineages entered into the general gene pool from the same demographic event. BATWING constructed clear modal trees for three HGs (F*-M89, L1-M27 and H1-M52) but not for the others (R1a1-M17, H-M69, J2-M172 and R2-M124). The three modal trees (Figure S3a–S3c) exhibited very diverse branching patterns with tribal and non-tribal MPGs being mixed randomly and without the outgroups corresponding to the MPG with the highest HG frequency, as would be expected if this MPG were the main source of this paternal lineage for other populations. Estimates of the time to most recent ancestor (TMRCA) for the HGs ranged from 11.4 Kya for F*-M89 to 6.1 Kya for L1-M27. Similar dates marking the founding of the clusters identified in the HG F*-M89 network with Ultranet clustering were obtained by BATWING using virtual UEPs to define clusters. The similar TMRCA estimates and the diverse tree topologies suggest that extant tribal and non-tribal groups derive from the ancient populations of the region, with population differentiation taking place at relatively similar times under complex demographic histories with multiple entries and sources of the common paternal lineages.

Finally, a third set of BATWING runs were performed using all HGs from individual populations within selected MPGs to test whether the grouping of these populations could have affected BATWING estimates of population divergence and phylogenetic relationships (Figure S4a–S4c). All endogamous populations grouped according to their MPG classification in the BATWING trees with the exception of the HTF-Irula clustering with other HTK tribes. This result was not unexpected because the Irula and the Kurumba were seen to share STR haplotypes in the F*-M89 and H*-M69 networks. BATWING estimated the differentiation between them to have occurred 3.4 Kya. In addition, BATWING assigned similar time frames to those in the previous two sets of runs, when major differentiation may have occurred among the endogamous populations, independently of the selected populations used. Moreover, the two most recent split estimates obtained by BATWING runs using endogamous DLF populations agrees with historical records, which indicate recent demographic expansions for the Vanniyars (2.3 Kya) and Nadars (1 Kya). These results further supported the classification of the seven MPGs, for which the population divergence time estimates were consistent for all sets of BATWING runs.

## Discussion

The study populations from Tamil Nadu were characterized by an overwhelming proportion of Y-chromosomal lineages that likely originated within India, suggesting a low genetic influence from western Eurasian migrations in the last 10 Kya. Although non-tribal groups exhibited a slightly higher proportion of non-autochthonous lineages than tribal populations, the common paternal lineages shared by TN populations are likely drawn from the same ancestral genetic pool that emerged in the late Pleistocene and early Holocene. We also noted that the current modes of subsistence have shaped the genetic structure of TN groups, with non-tribal populations being more genetically homogeneous than tribal populations likely due to differential levels of genetic isolation among them. Coalescence methods, employed to identify specific and distinctive periods when genetic differentiation among populations occurred, indicated a time scale of ∼6,000 years. We discuss below whether the timing of the male genetic differentiation of the populations fits better with archeological and historical records for the implementation of the Hindu Varna system or with agriculture expansions in the TN region.

### Endogamous social stratification preexisted the Varna system

Previous studies of Indian populations have grouped and analyzed the genetic data in the light of the Hindu Varna system [14], [15], [16] even though its origin and antiquity are still an ongoing topic of debate. One of the theories that has acquired wide support relates the establishment of the caste system to Indo-Aryan expansions from Western Eurasia into India around 3 Kya. An alternative view would see an earlier Indo-Aryan expansion with an introduction of cereal farming into Pakistan/North India around 8–7 kya. Genetic evidence reported by other studies that support these theories are mainly based on the high frequency of HG R1a1-M17 in Brahmin castes and their closer genetic affinity with West Eurasian populations compared to other Indian non-Brahmin castes and tribes [10], [20]. However, admixture analyses supporting a West Eurasian origin of the Brahmin may be biased due to the high frequencies of R1a1-M17 shared between these populations, as the rest of their Y-chromosomal variation shows little similarity [6], [7], [16]. Moreover, the recent discovery of new markers within R1a1-M17 has allowed Eastern European Y-lineages to be differentiated from those in Central/South Asia, locating the oldest expansion times with this lineage in Indus Valley populations, suggesting an earlier, possibly autochthonous origin of this HG in South Asia [68]. The Brahmin populations in the present study are also characterized by a significantly higher frequency of R1a1-M17 relative to other TN groups, but without any significant frequencies for HGs having a likely origin outside India. The TN Brahmin populations also present a very similar package of the most common HGs observed in 600 Brahmin individuals from all over India [16]. We noted that the highest STR variances for HG R1a1-M17 observed in SC and DLF, along with the lack of population-specific clusters in the R1a1-M17 network and the failure of BATWING to generate a definitive modal tree for this HG, all argue against the introduction of these paternal haplotypes through a single wave of Brahmin (i.e. Indo-Aryan) migration into the region.

Literary works from the Sangam period (300 BCE to 300 CE) describes a heterogeneous society that predates incorporation of already established populations into the Hindu Varna system [22] in TN. Ancient Tamil society was highly structured by habitat and occupation, where endogamy was practiced among populations known as *kudi* [37]. Many of the populations, such as the Valayar (meaning net weavers), Pulayar, Paliyan and Kadar, are cited in the Sangam literature using the same names that are employed today. Thus, a structured society practicing endogamy pre-existed in TN prior to the inferred arrival of the Indo-Aryans to this region. It is therefore most likely that the Varna system was superimposed on the pre-existing and historically attested social system without significant population transfer or input, implementing a new social hierarchy and order during the Pallava/Chola period from the 6^th^ through 12^th^ centuries CE [15], [22]. However, the implementation of the Varna system may have not been uniform across preexisting non-tribal populations since many of the populations within DLF and tribes do not practice either Vedic rituals or have very definite patrilineal system and clan exogamy. Overall, our results suggest that the genetic impact of Brahmin migrations into TN has been minimal and had no major effect on the establishment of the genetic structure currently detected in the region

### Models of agricultural expansions in the study region correlate with patterns of genetic diversity

The present study shows that the MPG classification reflects the genetic structure of the TN populations slightly better than other models, and that both tribal and non-tribal populations possess predominantly autochthonous lineages derived from a common gene pool established during the Late Pleistocene and Early Holocene. The distribution of over- and under-represented HGs suggests that populations within MPGs tend to share common genetic backgrounds. Using BATWING analysis, we estimate that social stratification for both tribal and non-tribal MPGs began between 6 Kya and 4 Kya, and detectable admixture between them has not occurred over the past 3 Kya, thereby allowing them to retain their genetic identity through cultural endogamy.

Both the overall Y-chromosomal HG distribution and the divergence estimates for tribal and non-tribal groups, are consistent with the archaeological dates and the demographic processes involved in the expansion of agriculture in South Asia. The South Deccan region near southern Karnataka and southwest Andhra Pradesh contains the earliest evidence for an integrated agro-pastoral system in South India, and likely acted as agricultural center and source of dispersion around 5 Kya [30], [31], [34], [69]. The genetic impact of the demographic processes involved during the development and spread of agriculture in India have been invoked under the Frontier theory framework [30]. According to this model, agricultural groups rapidly expanding into new environments suitable for farming created moving frontiers where autochthonous lineages from multiple pre-existing hunting and gathering forager populations were assimilated into the new agriculturalist populations, thereby producing centers of greater genetic diversity with less evidence of isolated evolution than observed in foraging populations. This mechanism was proposed by Semino *et al*, for convergence of multiple E-M123 founders into Turkey prior to re-expansion into Europe in order to explain the high diversity for that haplogroup [70]. The genetic patterns observed in this study, such as the presence of the oldest age estimates of autochthonous HGs found among the agriculturalist non-tribal populations (DLF), could reflect assimilated paternal lineages from genetically diverse pre-existing populations into common gene pools, as well as to suggest that today's tribal groups are not the sole source of these lineages.

In addition to this moving frontier, broader and more static agricultural frontier zones could also have arisen at later stages. In this area, stable and growing farming populations interacted with local foragers and created new cultural traditions, with some potential inter-marriage and assimilation through trade taking place. Southern Tamil Nadu and the Kerala zone represent one such agricultural frontier zone that has persisted to the present after local foragers began to adopt cultivation based on agricultural sedentism around 3 Kya [30]. Nowadays, TN tribes exhibit a wide variety of occupations and subsistence strategies, and mostly inhabit the Western Ghats Mountains, which harbor tropical and semi-tropical rain forests. In this context, two of the three tribal groups associated with foraging lifestyles (HTF and HTK) show the clearest signals of genetic drift, most likely due to strong founder effects and long-term isolation. They exhibit the lowest HG diversities (HTF: 0.687; HTK: 0.748), the highest proportion of putative autochthonous lineages (HTF: 95.3%; HTK: 88.5%), and the lowest ancestral effective population sizes estimated by BATWING (results not shown). In addition, the persistence of stronger genetic structure among HTF and HTK tribal populations, as seen in AMOVA, PCA and MDS analyses, suggests limited admixture with other TN populations. The absence of any human habitation sites in the Western Ghats until the Neolithic, and the late paleo-botanical evidence for cultivation, suggest a relatively late occupation of these mountains [34]. It is therefore possible that, upon agricultural expansion into previously non-cultivated areas, the present day tribal populations were displaced to more isolated regions, where they retained their mode of subsistence and genetic distinctiveness until the present day.

The overall Y-chromosomal landscape of TN suggests a complex process of agricultural expansion, which can be explained in terms of the formation of moving and static frontiers since 6 Kya, followed by migrations structured by habitat and occupation. However, because gene flow and differential assimilation of incoming migrations could alter the estimated divergence dates, they should be treated with caution. Our BATWING simulations and others from a previous study [62] have shown that topologies and population splits for modal trees are susceptible to admixture between already differentiated populations, which considerably reduces the times of split, but insensitive to migration into a region bringing new paternal lineages. This means that the divergence time estimates presented here likely reflect the latest major admixture that occurred among the populations being sampled from the TN region. In this regard, it is important to note that our BATWING estimates are concordant with historical records of major splits between two Vanniyar and between two Nadar populations, thereby supporting the ability of BATWING to detect recent demographic events. Thus, the main limitation of BATWING is related to its lack of power to detect earlier demographic events and its bias in clearly detecting recent gene flow among the populations studied. In any case, our conclusions supporting a common autochthonous Indian genetic heritage from the late Pleistocene/early Holocene for both tribal and non-tribal populations and refuting the hypothesis of the establishment of a structured and endogamous system due to an Indo-Aryan migration or implementation of the Varna System, still hold even if the BATWING divergence times are underestimates.

Although previous genetic studies have already drawn some of the conclusions presented here [6], [7], [16], [23], this is the first time (which we are aware of) that a genetic study showed clear evidences of the existence of long-standing endogamous population identities within a highly structured Indian society established prior to the regional implementation of the Varna system. Further, these paternal genetic identities likely resulted as a byproduct of demographic processes that occurred during the creation of moving and static frontiers of agricultural expansions into TN [30], [69]. The meticulous sampling strategy focused on a local area, and comparison of genetic data with the paleoclimatic, archeological, and historical background information available for the region, allowed us to address these questions at a deeper level than previous studies have. Moreover, this approach reduced considerably the confounding relationships among socio-cultural factors allowing us to further explore and test in detail the relationships between ethnography and genetics. Indeed, the pattern of long-term separation among populations within and between MPGs, and the genetic affinities of the constituent populations within MPGs, are significant features that would be lost if populations were pooled by other proxies based on broad classifications such as tribal versus non-tribal categories or Varna rank-caste hierarchy. We were also able to show that not all of the tribal populations reflect the oldest genetic legacy of the region and that each tribal population has a unique and distinct evolutionary history.

Thus, the sampling and analytical approach employed here suggest that detailed local genetic studies within India could give us new insights about the relative influences of past demographic events in relation to other socio-cultural and economic factors that might have influenced the population structure of the whole of India that is observed today. Nevertheless, it cannot be assumed that the same demographic processes or socio-cultural factors affected Indian populations from different regions in a similar manner. Whether corresponding Y chromosome genetic patterns can be also detected in other tribal and non-tribal populations within the South Deccan or in other Indian regions that have already been identified as centers of agricultural expansions, are open questions that future studies could potentially address using the methods presented here. Finally, it would also be important to investigate the relative impact of the processes explained here on the diversity patterns in other genomic regions by studying mtDNA and autosomal variation.

## Supporting Information

Figure S1 **PCA plot showing the first two principal components of haplogroup frequencies for 97 non-tribal (circles) and tribal (squares) populations of India and nearby regions from previous publications, compared to the non-tribal (horizontal ovals) and tribal (diamonds) populations from the present study.** Symbols have been colored according to linguistic classification. Population codes and references are shown in Table S3.
(TIF)

Figure S2 **Reduced median network of 17 microsatellite haplotypes within haplogroup.** (a) HG C-M130 using 74 chromosomes, (b) HG H1-M52 using 292 chromosomes (c) HG H- M69 using 79 chromosomes, (d) HG L1 – M27/M76 using 235 chromosomes, (e) HG R1a1-M17 using 214 chromosomes. Circles are colored based on the 7 Major Population Groups as shown in Figure 1, and the area is proportional to the frequency of the sampled haplotypes. Branch lengths between circles are proportional to the number of mutations separating haplotypes.
(TIFF)

Figure S3 **Modal tree obtained by BATWING indicating the coalescence time divergence estimates (in years) among Major Populations Groups (MPG) using 17 STRs from haplogroup (a) F-M89, (b) H1-M52, (c) L1-M26/M72.**
(TIFF)

Figure S4 **Modal tree obtained by BATWING indicating the coalescence time divergence estimates (in years) among endogamous populations within (a) HTF and HTK groups, (b) DLF, (c) BRH and HTC, using 17 STRs from all haplogroups.**
(TIFF)

Table S1 **List of Y chromosome SNPS and haplotype data for the 1680 individuals from 31 tribal and non-tribal populations presented in this study.**
(XLS)

Table S2 **AMOVA analysis of various population groupings based on the 17STR haplotype & 95%CI based on re-sampling of the samples across populations.**
(XLS)

Table S3 **List of population codes and their publication references used in Figure S1.**
(XLS)

Table S4 **Fishers exact test** ***p*** **-values for the NRY HG frequencies among the 7 Major Populations Groups and among the 31 sampled populations.**
(XLS)
