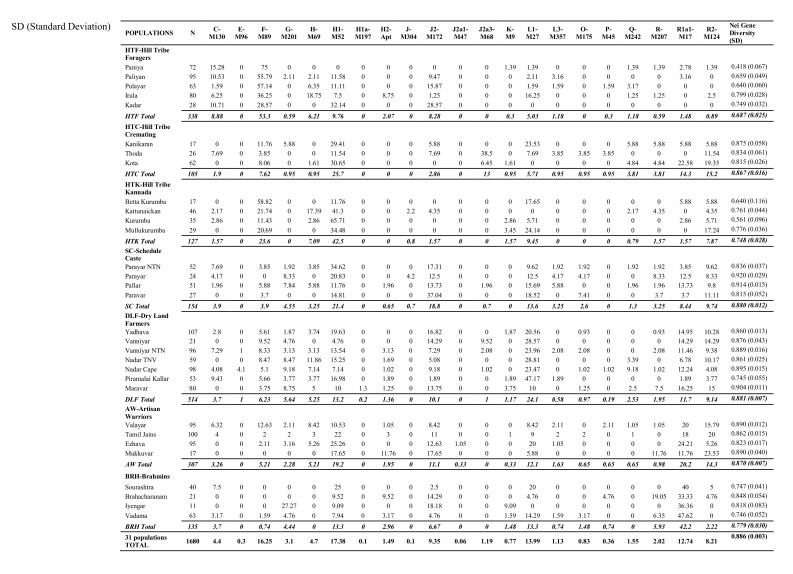# Correction: Population Differentiation of Southern Indian Male Lineages Correlates with Agricultural Expansions Predating the Caste System

**DOI:** 10.1371/annotation/8663819b-5ff0-4133-b70a-2d686dfb0a44

**Published:** 2013-07-26

**Authors:** GaneshPrasad ArunKumar, David F. Soria-Hernanz, Valampuri John Kavitha, Varatharajan Santhakumari Arun, Adhikarla Syama, Kumaran Samy Ashokan, Kavandanpatti Thangaraj Gandhirajan, Koothapuli Vijayakumar, Muthuswamy Narayanan, Mariakuttikan Jayalakshmi, Janet S. Ziegle, Ajay K. Royyuru, Laxmi Parida, R. Spencer Wells, Colin Renfrew, Theodore G. Schurr, Chris Tyler Smith, Daniel E. Platt, Ramasamy Pitchappan, The Genographic Consortium

Some of the values in Table 2 were included in the wrong columns. Please see the correct version of Table 2 at the following link: 

**Figure:**