# Supplementary figures and images for: Population Differentiation of Southern Indian Male Lineages Correlates with Agricultural Expansions Predating the Caste System
Source: PLoS One. 2012 Nov 28;7(11):e50269. doi: 10.1371/journal.pone.0050269 (PMC3508930; doi:10.1371/journal.pone.0050269)

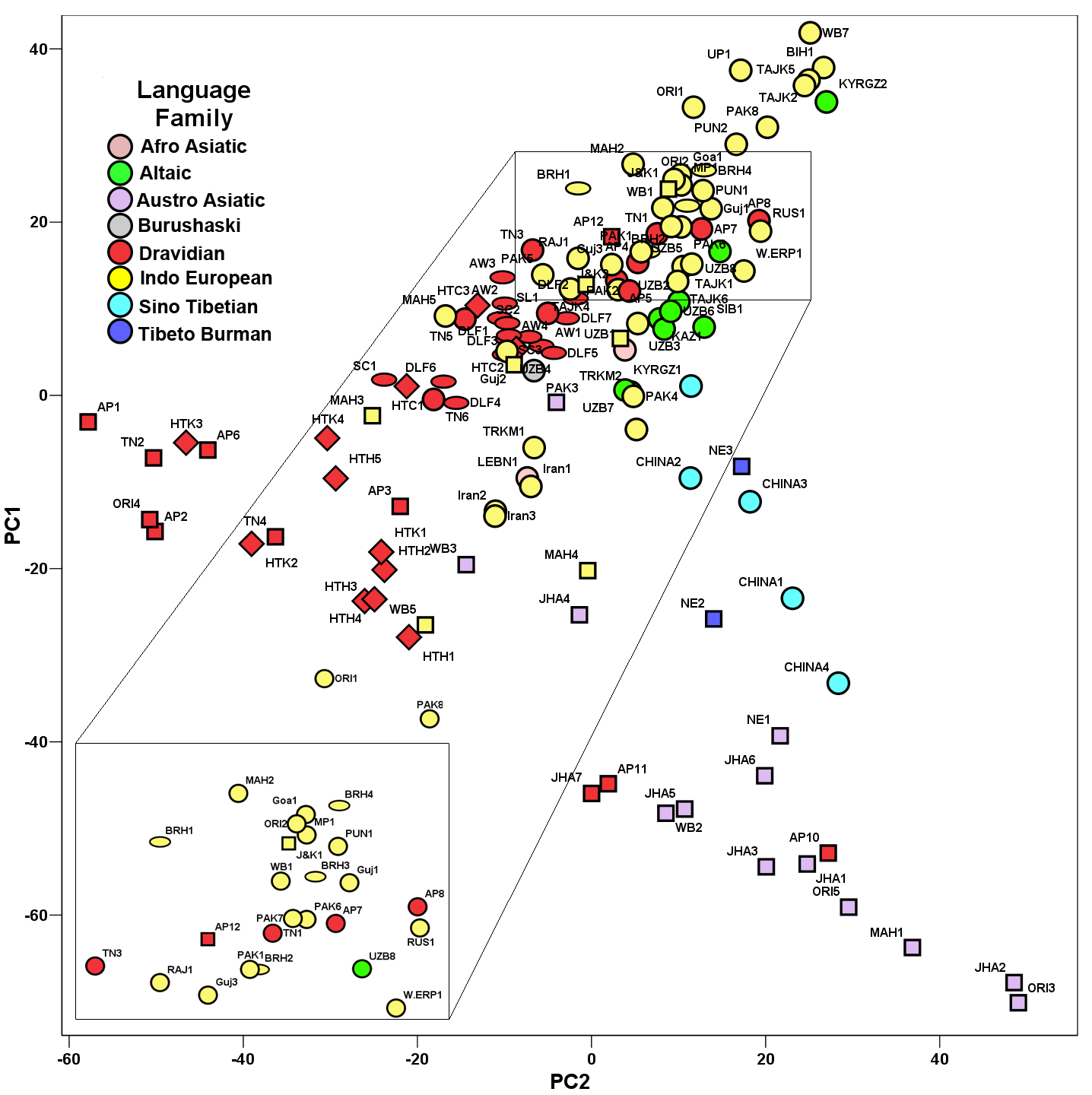

Supplement: Figure S1 — PCA plot showing the first two principal components of haplogroup frequencies for 97 non-tribal (circles) and tribal (squares) populations of India and nearby regions from previous publications, compared to the non-tribal (horizontal ovals) and tribal (diamonds) populations from the present study. Symbols have been colored according to linguistic classification. Population codes and references are shown in Table S3. (TIF) [file pone.0050269.s001.tif]

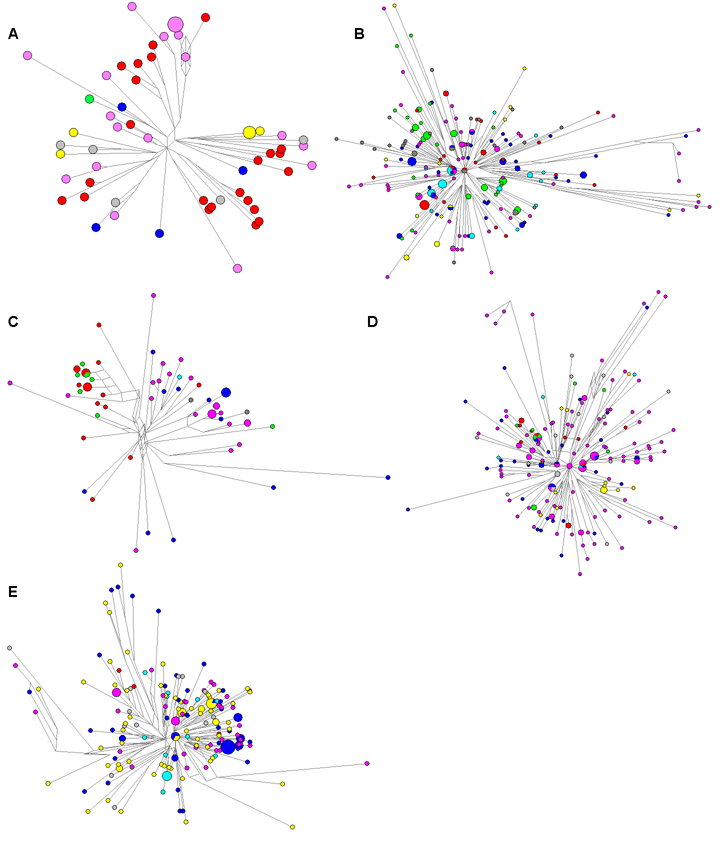

Supplement: Figure S2 — Reduced median network of 17 microsatellite haplotypes within haplogroup. (a) HG C-M130 using 74 chromosomes, (b) HG H1-M52 using 292 chromosomes (c) HG H- M69 using 79 chromosomes, (d) HG L1 – M27/M76 using 235 chromosomes, (e) HG R1a1-M17 using 214 chromosomes. Circles are colored based on the 7 Major Population Groups as shown in Figure 1, and the area is proportional to the frequency of the sampled haplotypes. Branch lengths between circles are proportional to the number of mutations separating haplotypes. (TIFF) [file pone.0050269.s002.tif]

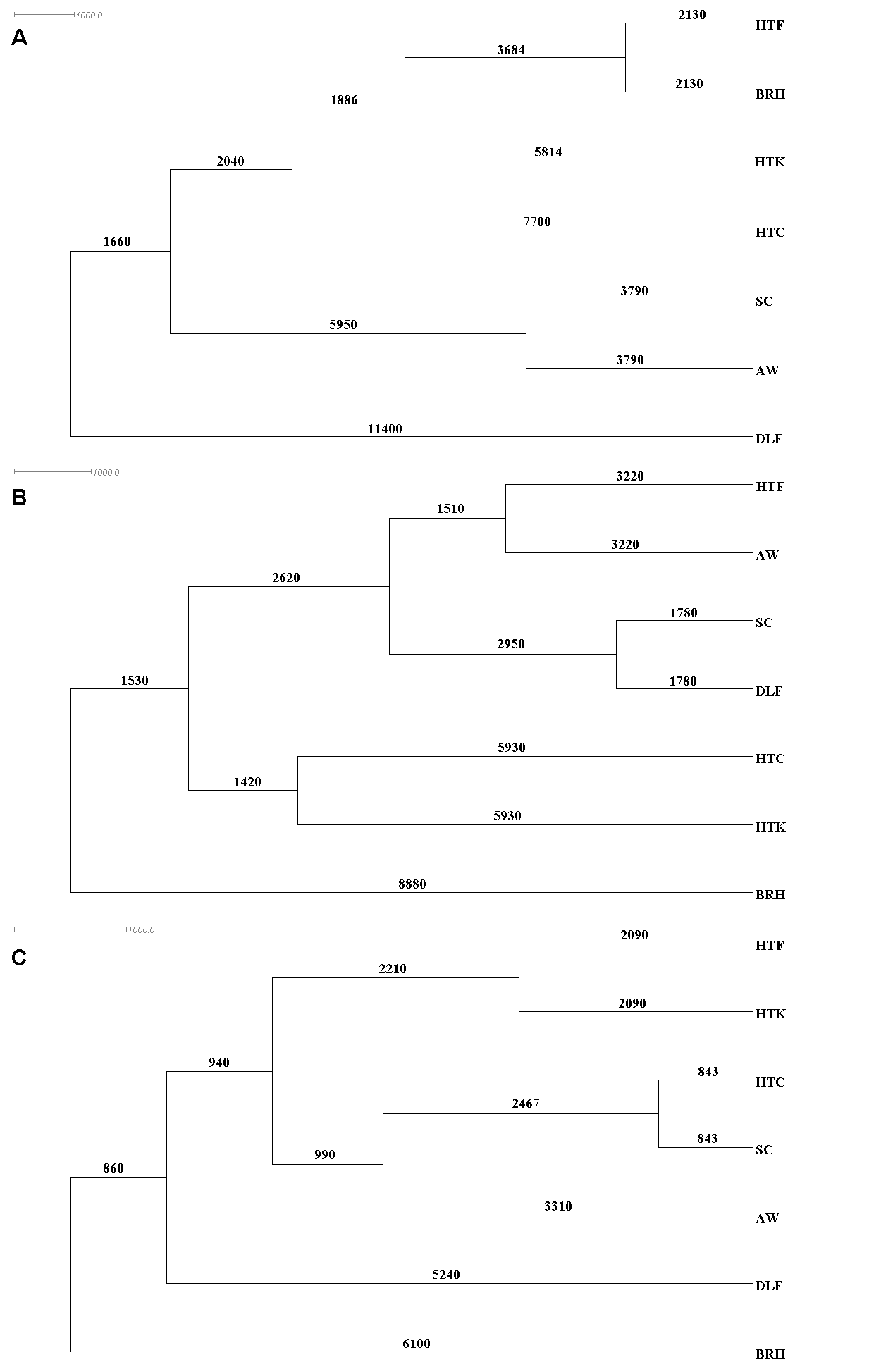

Supplement: Figure S3 — Modal tree obtained by BATWING indicating the coalescence time divergence estimates (in years) among Major Populations Groups (MPG) using 17 STRs from haplogroup (a) F-M89, (b) H1-M52, (c) L1-M26/M72. (TIFF) [file pone.0050269.s003.tiff]

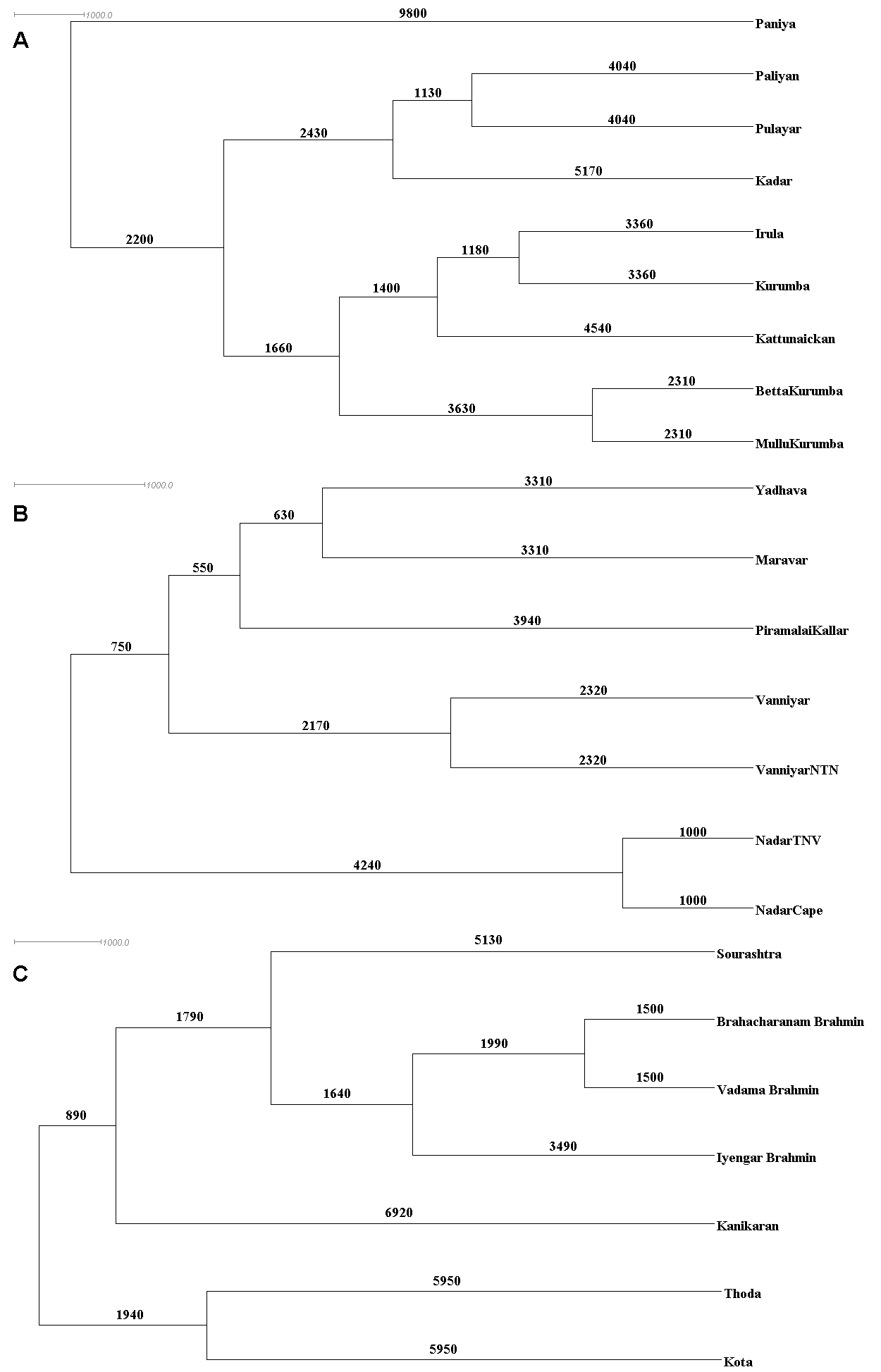

Supplement: Figure S4 — Modal tree obtained by BATWING indicating the coalescence time divergence estimates (in years) among endogamous populations within (a) HTF and HTK groups, (b) DLF, (c) BRH and HTC, using 17 STRs from all haplogroups. (TIFF) [file pone.0050269.s004.tiff]
